# Nodal ultrasound for regional recurrence detection in sentinel lymph node biopsy‐positive cutaneous melanoma patients undergoing cross‐sectional imaging

**DOI:** 10.1002/ski2.305

**Published:** 2023-10-15

**Authors:** Samvel Gyurdzhyan, Vijaytha Muralidharan, Lucy Y. Liu, John B. Sunwoo, Lisa C. Zaba, Susan M. Swetter

**Affiliations:** ^1^ Department of Dermatology Pigmented Lesion and Melanoma Program Stanford University Medical Center and Cancer Institute Stanford California USA; ^2^ Stanford University School of Medicine Stanford California USA; ^3^ Department of Otolaryngology – Head and Neck Surgery Stanford University School of Medicine Stanford California USA; ^4^ Dermatology Service Veterans Affairs Palo Alto Health Care System Palo Alto California USA

Dear Editor,

There are limited data comparing the efficacy of ultrasound (US) to cross‐sectional imaging (computed tomography [CT], positron emission tomography [PET]‐CT, or magnetic resonance imaging [MRI]) for detection of regional nodal recurrence in sentinel lymph node (SLN) biopsy‐positive (SLNB‐positive) cutaneous melanoma (CM) patients who forego completion lymph node dissection (CLND).^1^, ^2^ The seminal MSLT‐2 and DeCOG‐SLT trials for SLNB‐positive patients who underwent nodal US surveillance over CLND focused primarily on relapse free‐ and melanoma‐specific survival, rather than radiographic detection of regional nodal recurrence, and they predated the advent of effective adjuvant therapy.^3^, ^4^

While CM guidelines promote active surveillance with nodal US or other imaging over CLND,^5^ considerable variability exists in the United States regarding choice of imaging modality, patient adherence to nodal US schedules,^2^, ^6^ as well as radiologic criteria and expertise for nodal US detection of regional recurrence, with centres often relying on clinical lymph node examination and standard cross‐sectional imaging (CT or PET‐CT). Herein, we report our institutional experience of active nodal US surveillance and cross‐sectional imaging in a cohort of SLN‐positive patients who did not undergo CLND.

Following IRB approval, we retrospectively identified SLN‐positive patients ≥18 years of age from June 2011 through December 2019, who underwent nodal US surveillance in lieu of CLND and were followed for a minimum of 2 years or until recurrence/metastasis/death, through June 2022. Clinical, pathological, and radiologic details were collected (Table 1). Primary endpoints were US detection of regional nodal recurrence in comparison to clinical exam and/or cross‐sectional imaging (PET‐CT or CT). US schedule adherence was defined as ≥1 exam every 6 months, in accord with previously described surveillance schedules.^6^ Negative and positive predictive values (NPV, PPV) were determined using total number of US exams performed. True positive and false negative US findings for nodal recurrence were confirmed pathologically.

**TABLE 1:** Clinicopathologic characteristics of sentinel lymph node‐positive patients undergoing regional nodal ultrasound surveillance (*n* = 45).

Age, y, median (range)	58 (19–85)
Sex, *n* (%)	
Female	25 (55.6%)
Male	20 (44.4%)
Race, *n* (%)	
White	42 (93.3%)
Unknown	3 (6.7%)
Primary site	
Head and neck	8 (17.8%)
Upper limb	13 (28.9%)
Lower limb	10 (22.2%)
Trunk	14 (31.1%)
Histologic subtype	
Superficial spreading	21 (46.7%)
Acral lentiginous	6 (13.3%)
Lentigo maligna	6 (13.3%)
Nodular	10 (22.2%)
Desmoplastic	1 (2.2%)
Spindle	1 (2.2%)
Breslow depth, mm, median (range)	1.5 (0.75–17)
Ulceration, *n* (%)	12 (26.7%)
Mitotic rate, *n*/mm^2^, median (range)	2 (0–30)
Perineural invasion	2 (4.4%)
Lymphovascular invasion	3 (6.7%)
Extracapsular extension	3 (6.7%)
Microsatellites	2 (4.4%)
Satellite/in‐transit metastasis at diagnosis	3 (6.7%)
Number of positive SLNs, median (range)	1 (1–4)
SLN metastasis largest diameter, mm, median (range)	0.34 (0–10)
Adjuvant therapy	14 (18.4%)
Nivolumab	7 (9.2%)
Pembrolizumab	5 (6.6%)
Interferon	2 (4.4%)
Dabrafenib/Trametinib	0 (0%)

Abbreviation: SLN, Sentinel lymph node.

Forty‐five patients met inclusion criteria and underwent a median of 5 nodal US exams (range 2–10) that were recommended every 3–6 months after SLN biopsy, with median follow‐up of 42 months (range 24–115). Fourteen patients underwent nodal US at least every 6 months and 30 underwent less frequent imaging related in part to the COVID‐19 pandemic; 1 patient was lost to follow‐up. Fourteen patients received systemic adjuvant therapy (Table 1). There were no statistically significant differences between frequently and less frequently imaged (i.e. US adherent vs. US non‐adherent) cohorts based on patient or tumour characteristics. Categorical variables were compared using the Fisher's exact test, while the Kruskal‐Wallis test for independent samples was utilised for analysing continuous variables.

Melanoma recurrence/metastasis was observed in 11 patients (24.4%) including 6 on adjuvant immunotherapy, at median 18 months follow‐up (range 3–37), with overall recurrence‐free survival of 75.6%. Three patients (6.7%) had local (satellite/in‐transit) recurrence; 2 (4.4%) had regional nodal recurrence; 1 (2.2%) had local‐regional recurrence; 4 (8.9%) developed distant metastasis; and 1 (2.2%) developed local, regional, and distant metastasis.

Of the 4 patients with regional nodal recurrence (all US schedule adherent), 3 had AJCC‐8 stage IIIC melanoma (1 on adjuvant pembrolizumab) and 1 had stage IIIA disease, with first recurrence at median 12 months (range 5–18). Three (75%) regional recurrences were detected via surveillance US, and 1 (25%) was detected clinically (Table 2). All US‐detected regional nodal recurrences were metabolically active on concomitant PET‐CT. Of 214 total US exams, 10 revealed suspicious findings (3 true positive, 7 false positive) and 204 revealed no concerning findings (1 false negative, 203 true negative), resulting in PPV 30.0%, NPV 99.5%, sensitivity 75.0%, and specificity 96.7%.

**TABLE 2:** Clinicopathologic characteristics of sentinel lymph node‐positive patients with regional nodal recurrence (*n* = 4).

Regional nodal recurrences (*n* = 4)	
Number of ultrasound exams before nodal recurrence, median (range)	2.5 (1–4)
Number of clinical exams before nodal recurrence median (range)	7 (4–11)
Time to recurrence (months), median (range)	12 (5–18)

Clinicopathologic characteristics of sentinel lymph node‐positive patients with regional nodal recurrence				
Patient age (years), sex	70, F	74, M	70, F	66, F
Primary site	Head and neck	Lower limb	Lower limb	Trunk
BD (mm)	3.7	2.8	4.9	1.3
Ulceration present	No	Yes	Yes	No
MR (*n*/mm^2^)	14	3	5	1
LVI present	Yes	No	No	No
ECE present	No	No	Yes	No
AJCC‐8 stage	IIIC	IIIC	IIIC	IIIA
Nodes (positive/removed)	1/1	4/4	1/1	1/5
Largest metastasis (mm)	3.5	3.1	Isolated tumour cells	Isolated tumour cells
Adjuvant therapy	Pembrolizumab	NA	NA	NA
Number of nodal US	3	2	3	4
Method of nodal detection	Clinical	US	US	US
US Adherent^a^	Yes	Yes	Yes	Yes
Synchronous distant disease	Yes	No	No	No

Abbreviations: BD, Breslow depth; ECE, Extracapsular extension (present in one case due to several small extracapsular nests of atypical cells highlighted by S100 and SOX‐10 immunostains); LVI, Lymphovascular invasion; MR, Mitotic rate; NA, Not applicable; US, Ultrasound.

^a^ Ultrasound scheduling adherence defined as at least 1 nodal US every 6 months.

For SLNB‐positive CM, current NCCN guidelines recommend clinical observation without additional nodal surgery, but with mandatory radiographic nodal surveillance that includes nodal basin US surveillance (when radiologic expertise is available), or other imaging modalities (e.g. CT, MRI).5 Most treatment centres and SLNB‐positive patients opt for active surveillance over CLND, without significant differences in recurrence rates.^6^, ^7^, ^8^

Our findings suggest that while nodal US demonstrates high specificity in detecting regional recurrence, false positive findings are problematic, with clinical utility also limited by variability in radiologic protocols/interpretation and poor patient adherence to additional imaging. Routine cross sectional imaging for stage III patients with PET‐CT, though far costlier than US, revealed comparable nodal findings in our cohort; comparable data were not available for nodal recurrence detected via CT chest/abdomen/pelvis. Study limitations include retrospective assessment, small sample size, lack of uniformly‐applied radiologic criteria, and low adherence to nodal US schedules.

Within the context of modern (neo)adjuvant therapy associated with improved relapse‐free survival, prospective multi‐centre investigation with long‐term follow‐up is needed to determine whether routine cross‐sectional imaging is sufficient for detecting regional nodal recurrence in SLNB‐positive patients, and to define the optimal, cost‐effective, imaging modality/frequency that promotes patient adherence for radiologic surveillance.

## CONFLICT OF INTEREST STATEMENT

None to declare.

## AUTHOR CONTRIBUTIONS

**Samvel Gyurdzhyan**: Conceptualization (supporting); data curation (lead); formal analysis (equal); investigation (equal); methodology (equal); writing – original draft (equal); writing – review & editing (equal). **Vijaytha Muralidharan**: Data curation (equal); formal analysis (equal); investigation (equal); writing – original draft (equal); writing – review & editing (equal). **Lucy Y. Liu**: Conceptualization (supporting); data curation (equal); formal analysis (supporting); investigation (supporting); methodology (supporting); writing – original draft (supporting); writing – review & editing (supporting). **John B. Sunwoo**: Conceptualization (equal); investigation (equal); methodology (supporting); supervision (supporting); writing – original draft (supporting); writing – review & editing (supporting). **Lisa C. Zaba**: Conceptualization (supporting); investigation (supporting); methodology (supporting); supervision (supporting); writing – original draft (supporting); writing – review & editing (supporting). **Susan M. Swetter**: Conceptualization (lead); data curation (equal); formal analysis (equal); investigation (equal); methodology (equal); project administration (lead); resources (lead); supervision (lead); writing – original draft (equal); writing – review & editing (lead).

## FUNDING INFORMATION

This research received no specific grant from any funding agency in the public, commercial, or not‐for‐profit sectors.

## ETHICS STATEMENT

This study was approved by the Stanford University Institutional Review Board.

## Data Availability

The data that support the findings of this study are available from the corresponding author upon reasonable request.
